# Citation searching: a systematic review case study of multiple risk behaviour interventions

**DOI:** 10.1186/1471-2288-14-73

**Published:** 2014-06-03

**Authors:** Kath Wright, Su Golder, Rocio Rodriguez-Lopez

**Affiliations:** 1 Centre for Reviews and Dissemination, A/B Block, Alcuin College, University of York, York YO10 5DD, UK

**Keywords:** Systematic reviews, Information retrieval, Citation searching

## Abstract

**Background:**

The value of citation searches as part of the systematic review process is currently unknown. While the major guides to conducting systematic reviews state that citation searching should be carried out in addition to searching bibliographic databases there are still few studies in the literature that support this view. Rather than using a predefined search strategy to retrieve studies, citation searching uses known relevant papers to identify further papers.

**Methods:**

We describe a case study about the effectiveness of using the citation sources Google Scholar, Scopus, Web of Science and OVIDSP MEDLINE to identify records for inclusion in a systematic review.

We used the 40 included studies identified by traditional database searches from one systematic review of interventions for multiple risk behaviours. We searched for each of the included studies in the four citation sources to retrieve the details of all papers that have cited these studies.

We carried out two analyses; the first was to examine the overlap between the four citation sources to identify which citation tool was the most useful; the second was to investigate whether the citation searches identified any relevant records in addition to those retrieved by the original database searches.

**Results:**

The highest number of citations was retrieved from Google Scholar (1680), followed by Scopus (1173), then Web of Science (1095) and lastly OVIDSP (213). To retrieve all the records identified by the citation tracking searching all four resources was required. Google Scholar identified the highest number of unique citations.

The citation tracking identified 9 studies that met the review’s inclusion criteria. Eight of these had already been identified by the traditional databases searches and identified in the screening process while the ninth was not available in any of the databases when the original searches were carried out. It would, however, have been identified by two of the database search strategies if searches had been carried out later.

**Conclusions:**

Based on the results from this investigation, citation searching as a supplementary search method for systematic reviews may not be the best use of valuable time and resources. It would be useful to verify these findings in other reviews.

## Background

The main guides [1-4] to conducting literature searches for systematic reviews describe how citation searching can identify relevant papers and suggest that this approach should be carried out in addition to using comprehensive searches of bibliographic databases such as MEDLINE and Embase. There is, however, little evidence that this is an effective way to identify studies for potential inclusion in reviews. In particular, it is still uncertain whether citation searching can be used to identify unique studies not found by database searches or whether citation searching could be used to replace any of the existing database sources.

Citation searching may be hypothesized to be particularly useful to identify papers already retrieved by the searches but missed at the screening process due to an absence of immediate relevance indicated in the bibliographic records.

Systematic reviews traditionally rely upon extensive literature searching using multiple databases to ensure that all relevant studies are identified. It is usual to develop a highly sensitive search strategy to maximise the retrieval of relevant records although information specialists will aim for balance between sensitivity and precision to restrict, as far as possible, the number of irrelevant records retrieved. Even so, there is almost always a high proportion of records that do not meet the review’s inclusion criteria and will be rejected at an early stage of the review process. Additionally, when search results from the different databases are combined there is considerable duplication of records from across databases. When considering new resources or approaches to searching, such as Google Scholar, it is important to evaluate 1) sensitivity and precision of strategies 2) the resource’s potential for identifying unique studies not available in the databases already used and 3) time and cost.

Unlike database searching, citation searching starts with a known key paper, then identifies further potential papers by collecting the references that have cited the original paper. It assumes that if the first paper is relevant then later papers that cite the original paper may also be potentially relevant. In systematic reviews, the “key papers” used to carry out the citation searches will often be the studies that have been identified by the database searches and selected by the reviews team as meeting the inclusion criteria. Consequently, citation searches will need to be carried out after the main literature searching process has taken place.

Citation searching (sometimes called forward citation tracking), reference checking (examining the reference list at the end of a published paper) and contacting experts are all approaches used to identify evidence in addition to database searching [5]. Other techniques, “pearl growing” and “snowballing” where one relevant item is used to obtain others can also be used in systematic reviews [6] although these are not always described in sufficient detail to be able to replicate the steps in the process.

Few studies in the published literature examine the role of citation searching as a method of identifying studies for systematic reviews or describe in detail how and when it should be done. One study [7] audited the sources used in a systematic review of complex evidence i.e. the diffusion of service-level innovations in healthcare organisations. In this analysis, citation tracking using Science Citation Index (SCI), Social Science Citation Index (SSCI) and Arts and Humanities Citation Index (AHCI) identified 26 unique studies that constituted 12% of the empirical studies included in the final review. The authors tracked “selected papers” but do not report how many papers were selected for citation tracking or how the selection was made. This approach was considered to be an “important search method” especially as it successfully identified systematic reviews that had been published in less mainstream journals with three reviews not being identified by the usual database searching methods.

Another paper [5] compared the merits of different search techniques in retrieving evidence in the social sciences literature. Citation searching was one of a range of techniques that was evaluated, the others being traditional database searching, reference checking, contact with experts and pearl growing. The case study systematic review was a systematic review of qualitative studies investigating how to enhance the student experience of workplace based e-learning. In this instance the citation tracking tools used were Google Scholar, Science Citation Index (SCI), Social Science Citation Index (SSCI) and CINAHL. The citation tracking exercise was carried out after the database searching was performed and the studies had been selected for inclusion. It reports that the 30 included studies identified by the traditional database search produced a total of 75 citations after deduplication from a combination of the four citation sources used. The authors do not report the number of citations retrieved from each of the tracking tools or which of them identified the most citations. 14 of the citations were considered to be potentially relevant with 11 being uniquely identified by the citation searching. Three of these 11 were included in the review.

Although citation searching may identify relevant papers, it is also important to investigate whether the papers identified are unique or whether the additional records simply duplicate papers that have already been retrieved by the original searches. Citation searching represents a significant additional investment of searching effort and could also introduce a delay into the systematic review process so it is important to assess its ability to identify unique material.

The value of citation searching may also be in providing the context of the paper or a more useful insight into a paper’s potential relevance than can be obtained when screening the title and abstract alone.

Although many different citation searching databases are available their relative value has not been extensively evaluated and which citation searching database provides the most references is still unknown. Several studies have focused on investigating the performance of Google Scholar’s citation tracking feature as compared with similar features offered by other established resources such as Scopus and Web of Science. One study [8] took a sample of 30 publications written by nursing academics and compared how often they were cited by other publications according to CINAHL, Scopus, Web of Science and Google Scholar. The results of the investigation showed that the different databases found both unique citations and duplicated references. Another study [9], compared citation counts for articles from two topic areas (oncology and condensed matter physics) in two different years. Their findings showed that each of the tools identified unique material but the performance of each depended upon the topic and the subject year. Consequently, researchers who wish to have a comprehensive picture of their research impact would need to use more than one citation tracking tool.

To date, there is limited research on the value of citation searching for systematic reviews and, while some studies have investigated the coverage of Google Scholar, no consensus has been reached.

The objectives of this study are to investigate (1) the overlap between the four citation sources to identify which citation tool, or combination of tools, is the most useful to use and (2) whether citation searching identified any relevant records in addition to those retrieved by the original database searches.

The case study is based on a scoping review evaluating any intervention targeting change in at least two risk behaviours (e.g. smoking, alcohol misuse, physical inactivity, unhealthy diet, illicit drug use, sexual risk behaviour, lack of seat belt use, lack of motorcycle/bicycle helmet use, lack of sunscreen use, gambling, poor oral hygiene and drink driving). The full search strategies are available in the Additional file 1 and the published report, *A scoping review of multiple risk behaviour interventions,* will be available on the Public Health Research Consortium website http://phrc.lshtm.ac.uk/project_2011-2016_002.html.

The literature searches for the project were developed using MEDLINE via OVIDSP and subsequently translated for use with the other databases. The MEDLINE search strategy was complex and lengthy – 254 lines in total. One section of the search strategy used synonyms and variants of “multiple risk behaviours” and “lifestyle modifications” to identify relevant studies. An earlier project, *A systematic review on the clustering and co-occurrence of multiple risk behaviours in the UK,* that will also be available from the PHRC website had, however, identified that this approach was inadequate to identify all potentially relevant studies so the search strategy also included search terms for specific named behaviours such as alcohol misuse, physical inactivity, unhealthy diet and so forth. To retrieve studies that referred to two or more of these risk behaviours the search terms were initially combined using the Boolean AND operator (smoking AND physical activity; smoking AND unhealthy diet; smoking AND illegal drug use etc.) with the resulting search sets being then combined using the Boolean OR. The search strategy also included filters to restrict the results to various study designs e.g. trial, evaluation study, before and after studies and interrupted time series and also to various types of setting e.g. workplaces, communities. This approach was replicated for the search strategies used in the Embase and PsycINFO databases. For ASSIA, CENTRAL and Science Citation Index, however, it was difficult to construct comparable search strategies because of differences in the database search interfaces. Consequently, for these three databases, the search strategy consisted solely of search terms for “multiple risk behaviours” and “interventions, programmes” and “change”.

The searches identified 21,835 records after deduplication for sifting by the project’s review team.

## Methods

We carried out two analyses; the first examined the overlap between the four citation sources to identify which citation tool, or combination of citation tools, was the most useful to use and the second investigated whether the citation searches had identified any relevant records, particularly in addition to those retrieved by the original database searches.

We used the 40 included studies [10-49] identified by the traditional database searching from the case study scoping review of interventions for multiple risk behaviours and searched for each of them in the four citation sources of interest – Google Scholar, Scopus, Web of Science (WoS) citation searches and OVIDSP MEDLINE. We retrieved the details of all the papers that cited the studies, and downloaded the citations into bibliographic software.

Two researchers (KW and SG) then grouped records to enable identification of records available from all four resources, from three of the resources, from two of the resources, and unique records in order to assess the comparative value of the citation sources.

For the second analysis we compared the performance of the citation tracking sources, singly and in combination, with the performance of the database search strategies in order to assess the value of citation searching in the systematic review process. We imported the 1789 records identified from the citation searching into another Endnote library and recorded our inclusion/exclusion decisions.

Before scanning the citation tracking records for potential included studies we had planned to remove any records already identified by the database searching. However, many of the records downloaded from the citation resources were of much poorer quality than the bibliographic records downloaded from databases. Some very brief records consisted of authors’ names and short title only e.g. “phase one”, “Panel 6”, “letters” and so on. We were uncertain whether the automated deduplication processes using variants of the deduplicating algorithm within the bibliographic software would reliably identify duplicates. Consequently, we decided that, rather than attempt to deduplicate the whole set of records, we would restrict the deduplication to the smaller number of potentially included records. Consequently, the deduplication took place much later in the process than would usually be the case in the systematic reviews process.

Two researchers (KW and RL-R) scanned the 1789 records to assess whether or not they met the inclusion criteria for the review. The records were initially coded as 1 = Yes, 2 = No, 3 = Maybe, 4 = not enough information. There were 99 category 4 records: of these, some did not include an abstract, others were in a language other than English, or the downloaded record was incomplete. Where possible, we did further searching to identify additional information that would allow us to make a decision about the record’s potential inclusion. Although the amount of time required to find this additional information was significant, it did reduce the number of these records from 99 to 15. After the initial sift, 61 of the 1789 records (53 category 1 records and 8 category 3 records) were considered to be potential included studies.

Deduplicating the 61 records against the records already identified by the database searches identified 35 of the records as duplicates of the original search results and these were then excluded from the process. The remaining 26 records were then considered for inclusion in the review by a second team of researchers.

We used 1) the total number of included studies (40) and 2) the total number of records retrieved per database/citation tracking resource and 3) the number of included studies retrieved per database/citation tracking resource, to calculate the sensitivity, precision and number needed to read (NNR) for each of the database search strategies, for each of the citation tracking resources and for the citation tracking resources combined.

Sensitivity % is calculated using the following formula:

$$\frac{\;\text{Number}\;\text{of}\;\text{included}\;\text{records}\;\text{retrieved}\;}{\text{Total}\;\text{number}\;\text{of}\;\text{included}\;\text{records}}\times \;100$$

Sensitivity indicates the ability of the strategy to retrieve relevant records and, for a systematic review, a high level of sensitivity is required to ensure as few potentially relevant records as possible are missed. Conversely, search strategies with a lower sensitivity will miss a high proportion of relevant articles.

The precision values for the search strategies were calculated using the following formula:

$$\frac{\text{Number}\;\text{of}\;\text{included}\;\text{records}\;\text{retrieved}}{\text{Total}\;\text{number}\;\text{of}\;\text{records}\;\text{retrieved}}\times \;100$$

The number needed to read (NNR) is a measure of how many papers in the set need to be read before one relevant paper is identified. It was calculated using the following formula:

$$\frac{\text{Total}\;\text{number}\;\text{of}\;\text{records}\;\text{retrieved}}{\text{Number}\;\text{of}\;\text{included}\;\text{records}\;\text{retrieved}}$$

or the inverse of precision.

## Results

### Citation tracking resources

The total number of records identified from all citation sources was 4161 and after deduplicating there were 1789 records. Google Scholar identified the greatest number of citations - 1680, followed by Scopus at 1173, Web of Science at 1095, and OVIDSP MEDLINE at 213 (Table 1).

**Table 1:** Records identified by each of the 4 citation resources

	**Google scholar**	**Scopus**	**Web of science**	**OVIDSP**
Total records	1680	1173	1095	213
Unique records	558	71	68	5

The highest number of unique records were identified by Google Scholar (558) followed by Scopus (71), Web of Science (WoS) (68) and OVIDSP MEDLINE (5). Each of the resources did, therefore, contribute some unique records to the total number of citation records: Scopus (3.96%), Web of Science (3.8%) OVIDSP MEDLINE (0.27%) (Table 2).

**Table 2:** Best retrieval rate using 2 of the 4 citation resources

**Google scholar & Scopus**	**Google scholar & WoS**	**Google scholar & OVIDSP**	**Scopus & WoS**	**Scopus & OVIDSP**	**WoS & OVIDSP**
1716	1712	1547	1224	1103	1035

The highest number of records retrieved from just 3 sources would have been from using Google Scholar, Scopus and Web of Science (1784 records). If only two sources could be searched, the highest retrieval rate would be achieved from using Google Scholar and Scopus (1716). A small number of records (150) were common to all 4 of the resources with the majority of the records being available in more than one resource (Table 3).

**Table 3:** Best retrieval rate using 3 of the 4 citation resources

**Google scholar, Scopus & WoS**	**Google scholar, Scopus & OVIDSP**	**Google scholar, WoS & OVIDSP**	**Scopus, WoS & OVIDSP**
1784	1721	1718	1231

Carrying out the citation searching added approximately 5 days of time to the overall project. Approximately 2 days were spent in downloading the 1680 records from Google Scholar, one day in downloading records from the other 3 resources and a further two days in screening all the citation records.

### Performance of database searching compared with citation tracking

The database searches of ASSIA, CENTRAL, Embase, MEDLINE, PsycINFO and Science Citation Index (SCI) identified 36,393 records (21,835 after deduplication) and 40 of these were selected for inclusion in the review. The highest sensitivity was achieved in MEDLINE 75% (30), followed by 62.5% (25) Embase, then 52.5% (21) in PsycINFO. The sensitivity of the other database searches was low - the lowest being ASSIA with 10% (4).

The precision values for the database searches were also low ranging from 0.17% for Science Citation Index (SCI) to 10.04% for CENTRAL. The NNR was particularly high for Science Citation Index (587) and Embase (527) while the NNRs for MEDLINE, PsycINFO and ASSIA were broadly similar (276,261 and 220). The lowest NNR was CENTRAL (96).

We calculated the precision and sensitivity of the citation tracking after deduplication. For Google Scholar, Scopus and Web of Science the sensitivity of the citation tracking was 20% while the sensitivity of OVIDSP MEDLINE’s citation tracking was much lower at 5%. Combining the results for all four citation sources gave a measure of sensitivity of 22.5%. The precision of the citation tracking was low for all the sources used ranging from 0.48% (Google Scholar) to 0.94% (OVIDSP MEDLINE). The overall precision of the citation searches was 0.5%. The NNR for Google Scholar was 210, with 147 and 137 for Scopus and Web of Science.

The full range of performance measures for both the databases and the citation tracking resources is presented in Table 4.

**Table 4:** Records identified by each of the bibliographic databases and citation tracking resources

**Database**	**Total records identified**	**Total included studies available**	**Included studies retrieved**	**Recall % (n = 40)**	**Precision %**	**Number needed to read (NNR)**
MEDLINE	8279	39	30	75%	0.36%	276
Embase	13176	38	25	62.5%	0.19%	527
PsycINFO	5475	29	21	52.5%	0.38%	261
SCI	7048	40	12	30%	0.17%	587
CENTRAL	1059	34	11	27.5%	1.04%	96
Google scholar	1680	38	8	20%	0.48%	210
Scopus	1173	39	8	20%	0.68%	147
Web of Science	1095	40	8	20%	0.73%	137
ASSIA	881	16	4	10%	0.45%	220
OVIDSP MEDLINE	213	39	2	5%	0.94%	107
Citation searching all sources	1789	40	9	22.5%	0.5%	199

### Unique studies identified by the citation tracking

The initial sift of the 1789 citation tracking records produced 26 potentially new studies after deduplication. From these, one additional study [50] was selected for inclusion in the scoping review that had not been identified by the traditional database searches of MEDLINE, Embase, PsycINFO, Science Citation Index, ASSIA and CENTRAL undertaken during the period 15^th^ January to 18^th^ January 2013.

We carried out further checks to confirm 1) whether the record was available in each of the original databases used, and 2) whether it would have been available when the original searches had been undertaken. We discovered that a record for the paper [50] was not available in ASSIA, CENTRAL, MEDLINE (OVIDSP), or PsycINFO on the date of the check (15^th^ November 2013). The record was available in Embase having been added on 5^th^ August 2013. While the record was available in the SCI database when checked we were unable to find out the entry date as this information is not provided by the database producer. The additional study would have been identified in both Embase and SCI databases by searches carried out at a later date.

## Discussion

To identify all the records in the citation set required a search for the included studies using all four citation resources.

The highest number of unique citations was identified by Google Scholar, followed by Scopus, then Web of Science. If only one of the citation tracking resources were available for use, Google Scholar would identify 93.9% (1680) of the records; if two were available then using Google Scholar plus Scopus would be the most fruitful (95.92%, 1716 records). Using 3 of the resources (Google Scholar, Scopus, and Web of Science) could identify a high percentage 99.7% (1784) of the total citations.

The citation feature of MEDLINE (OVIDSP) did identify one of the included studies that none of the other citation tracking resources retrieved. The coverage of MEDLINE (OVIDSP)’s citation feature is restricted to those 3,000 plus journals that are included in the journals@Ovid database of full text journals available from OVID.The analysis demonstrates the relatively higher number of citations available from Google Scholar and the potential value of Google Scholar for citation tracking, especially as it is a freely available resource unlike the subscription only products Scopus and Science Citation Index (SCI). References from websites and grey literature included in Google Scholar can, however, be poor quality with consequent limited value. In addition, Google Scholar doesn’t have the facility to easily and quickly download records into bibliographic software so can add to the time required. Routinely incorporating citation searching using any of the available products into the systematic review process would add to the overall time required as this process can only be conducted after the database searches have been carried out and the included studies identified. Using Google Scholar significantly increased the time spent in downloading records as there is no batch export facility so each of the 1680 records had to be downloaded individually. This is a barrier to routinely using Google Scholar to carry out citation searching. Other citation resources with more sophisticated features are currently easier and quicker to use.

The second analysis focused on whether the citation search results identified any further relevant records in addition to those retrieved by the original database searches. The sensitivity of the database searches carried out for this scoping review, ranging from 10% to 75% was low when compared with the usual sensitivity values for searches carried out for systematic reviews. The highest sensitivity values achieved were MEDLINE at 75% and EMBASE at 62.5% while the search strategy used for ASSIA had the lowest value at 10%. It is worth noting that, while all 40 of the included studies were available in Science Citation Index (SCI), the search strategy only identified 12 of them. The very low sensitivity values for ASSIA, CENTRAL and Science Citation Index (SCI) could be attributed to the use of the abridged search strategy as well as the difficulty of the search topic i.e. evaluations of interventions targeting change in at least two risk behaviours.

The overall sensitivity value of the citation searching was 22.5%, with three of the resources (Google Scholar, Scopus and Web of Science) having identical sensitivity values of 20%. The overall precision value of the citation searches (0.5%) was higher than that of the majority of the databases searches. The performance of the Google Scholar citation searches in terms of the Number Needed to Read was broadly similar to that of some of the databases. The NNR for Google Scholar was 210 compared with 276 for MEDLINE, 261 for PsycINFO and 220 for ASSIA.

Combining citation searching with a search of just one database would have slightly increased the number of included studies identified. Using citation searching and MEDLINE would have identified 80% (32) of the included studies while using citation searching in addition to EMBASE would have identified 28 of the 40 included studies.

Citation tracking seems to perform well when measured using the NNR but it did identify only 9 of the 40 studies that met the review’s inclusion criteria. Eight of these had already been identified by the traditional databases searches while the ninth was not available in any of the databases when the original searches were carried out. It would, however, have been identified by two of the database search strategies if searches had been carried out at a later date.

### Limitations of this study

In terms of searching, the scoping review had very broad coverage. Its aim was to identify any intervention promoting change in at least two risk behaviours and the search strategy incorporated terms for all of these (smoking, alcohol misuse, physical inactivity, unhealthy diet, illicit drug use, sexual risk behaviour, lack of seat belt use, lack of motorcycle/bicycle helmet use, lack of sunscreen use, gambling, poor oral hygiene and drink driving) in various set combinations. The resulting complexity will almost certainly have had an impact upon the overall performance of the database search strategies. As with any case study, there is uncertainty about how far the results of this study can be generalised, especially to other reviews with a more restrictive focus.

## Conclusion

Google Scholar performed well in terms of the numbers of citations retrieved, and the number of unique citations retrieved. For this case study scoping review, the citation searches of Google Scholar, Web of Science, Scopus and OVIDSP MEDLINE identified one additional study for inclusion in the review that had not been identified by searching bibliographic databases. On the other hand, the citation searches only identified 9 of the studies that had been identified by the traditional database searching. Based on the results from this investigation, it seems that citation searching, as a supplementary search method for systematic reviews, may not be the best use of valuable time and resources.

## Competing interests

The authors declare that they have no competing interests.

## Authors’ contributions

KW conceived of the study, participated in the analysis, wrote the first and subsequent drafts of the manuscript. SG participated in the analysis; contributed to subsequent drafts of the manuscript and commented on the final version of the manuscript. RR-L participated in the analysis and commented on the final version of the manuscript. All authors read and approved the final manuscript.

## Pre-publication history

The pre-publication history for this paper can be accessed here:

http://www.biomedcentral.com/1471-2288/14/73/prepub

## Supplementary Material

Additional file 1 Database search strategies. Click here for file
