# Supplementary material for: Citation searching: a systematic review case study of multiple risk behaviour interventions
Source: BMC Med Res Methodol. 2014 Jun 3;14:73. doi: 10.1186/1471-2288-14-73 (PMC4048585; doi:10.1186/1471-2288-14-73)
Supplement: Additional file 1 — Database search strategies. [file 1471-2288-14-73-S1.docx]

**ASSIA**

**Via ProQuest**

**Search date 18^th^ January 2013**

**881 records identified**

[(all("behavior* change" NEAR/4 intervention*) OR all("behavior* change" NEAR/4 program*) OR all("behaviour* change" NEAR/4 intervention*) OR all("behaviour* change" NEAR/4 program*) AND pd(>19900101)) OR (all("multiple risk factor" NEAR/4 program*) OR all("multiple risk factor" NEAR/2 intervention*) OR all("multifactorial lifestyle" NEAR/2 program*) OR all("multifactorial lifestyle" NEAR/2 intervention*) AND pd(>19900101)) OR (all("health behavior*" NEAR/4 program*) OR all("health behavior*" NEAR/4 intervention*) OR all("health behaviour*" NEAR/4 program*) OR all("health behaviour*" NEAR/4 intervention*) AND pd(>19900101)) OR (all("multiple health behavior* change intervention*") OR all("multiple health behaviour* change intervention*") OR all("multiple behavior* risk factor* intervention*") OR all("multiple behaviour* risk factor* intervention*") OR all("multiple behavior* risk factor* program*") OR all("multiple behaviour* risk factor* program*") OR all("multiple risk behaviour* intervention*") OR all("multiple risk behavior* intervention*") OR all("multiple risk behaviour* program*") OR all("multiple risk behavior* program*") AND pd(>19900101)) OR (all(lifestyle NEAR/2 intervention*) OR all(lifestyle NEAR/2 program*) OR all("life style" NEAR/2 intervention*) OR all("life style" NEAR/2 program*) AND pd(>19900101))](http://search.proquest.com/myresearch/savedsearches.checkdbssearchlink:rerunsearch/327417/SavedSearches?site=assia&t:ac=SavedSearches)

**CENTRAL**

**Via Wiley**

**Search date 18^th^ January 2013**

**1059 records identified**

#1 lifestyle near/2 intervention*:ti,ab,kw or lifestyle near/2 program*:ti,ab,kw or "life style" near/2 intervention*:ti,ab,kw or "life style" near/2 program*:ti,ab,kw from 1990 to 2013, in Trials

#2 "behavior* change" next intervention*:ti,ab,kw or "behavior* change" next program*:ti,ab,kw or "behaviour* change" next intervention*:ti,ab,kw or "behaviour* change" next program*:ti,ab,kw from 1990 to 2013, in Trials (Word variations have been searched)

#3 "multiple risk factor" near/2 program*:ti,ab,kw or "multiple risk factor" near/2 intervention*:ti,ab,kw or "multifactorial lifestyle" next intervention*:ti,ab,kw or "multifactorial lifestyle" next program*:ti,ab,kw from 1990 to 2013, in Trials (Word variations have been searched)

#4 "health behavior*" next program*:ti,ab,kw or "health behavior*" next intervention*:ti,ab,kw or "health behaviour*" next program*:ti,ab,kw or "health behaviour*" next intervention*:ti,ab,kw from 1990 to 2013, in Trials (Word variations have been searched)

#5 "multiple health behavior* change intervention*":ti,ab,kw or "multiple health behaviour* change intervention*":ti,ab,kw or "multiple behavior* risk factor* intervention*":ti,ab,kw or "multiple behaviour* risk factor* intervention*":ti,ab,kw or "multiple behavior* risk factor* program*":ti,ab,kw from 1990 to 2013, in Trials (Word variations have been searched)

#6 "multiple behaviour* risk factor* program*":ti,ab,kw or "multiple risk behaviour* intervention*":ti,ab,kw or "multiple risk behaviour* program*":ti,ab,kw or "multiple risk behavior* intervention*":ti,ab,kw or "multiple risk behavior* program*":ti,ab,kw from 1990 to 2013, in Trials (Word variations have been searched)

#7 #1 or #2 or #3 or #4 or #5 or #6

**Embase**

**Via OVIDSP**

**Search date 15^th^ January 2013**

**13,176 records identified**

1 (health$ adj2 (diet$ or eating or food or foods)).ti,ab. (13569)

2 (unhealth$ adj2 (diet$ or eating or food or foods)).ti,ab. (1543)

3 fruit/ or vegetable/ (43569)

4 (fruit$ adj2 (eat or eats or eating or intake or consum$ or increas$ or portion$ or serving$ or frequenc$ or number$ or preference$ or choice$)).ti,ab. (6927)

5 (vegetable$ adj2 (eat or eats or eating or intake or consum$ or increas$ or portion$ or serving$ or frequenc$ or number$ or preference$ or choice$)).ti,ab. (6322)

6 "5 a day".ti,ab. (164)

7 "five a day".ti,ab. (40)

8 (junk food or fast food).ti,ab. (1914)

9 ((decreas$ or reduc$ or discourag$ or limit$ or lessen or eat$ less) adj2 (salt or fat)).ti,ab. (11916)

10 (food adj (choice$ or frequenc$ or select$)).ti,ab. (10976)

11 Feeding Behavior/ (49787)

12 eating habit/ or food preference/ (13695)

13 diet therapy/ (43181)

14 obesity/ (209708)

15 or/1-14 (359168)

16 (editorial or letter).pt. (1221865)

17 15 not 16 (341579)

18 limit 17 to yr="1990 -Current" (288201)

19 (physical$ adj3 (fit$ or train$ or activ$ or inactiv$ or endur$)).ti,ab. (81313)

20 (exercis$ adj3 (fit$ or train$ or activ$ or endur$)).ti,ab. (26417)

21 ((promot$ or uptak$ or encourag$ or increas$ or start$ or adher$) adj3 (exercis$ or gym$ or sport$ or fitness)).ti,ab. (24891)

22 ((decreas$ or reduc$ or discourag$) adj3 (sedentary or deskbound or desk-bound)).ti,ab. (537)

23 (sedentary behaviour$ or sedentary behavior$ or sedentary lifestyle$ or sedentariness).ti,ab. (4095)

24 sedentary lifestyle/ (2495)

25 ((watch$ or view$) adj2 (tv or television)).ti,ab. (3213)

26 (sport$ or walk$ or running or jogging or bicycling or biking or swimming).ti,ab. (179917)

27 (active adj (travel$ or transport$ or commut$)).ti,ab. (6569)

28 fitness/ or physical activity/ (85130)

29 exp Recreation/ or leisure/ (35054)

30 physical exercise/ (159644)

31 running/ or jogging/ or swimming/ or walking/ (56946)

32 or/19-31 (456811)

33 (editorial or comment).pt. (419336)

34 32 not 33 (449410)

35 limit 34 to yr="1990 -Current" (369145)

36 exp smoking/ (158022)

37 (smoking or antismoking or anti-smoking).ti,ab. (166408)

38 (smoker or smokers).ti,ab. (66367)

39 tobacco abuse/ or tobacco addiction/ or tobacco dependence/ (11329)

40 36 or 37 or 38 or 39 (242549)

41 (editorial or comment).pt. (419336)

42 40 not 41 (238083)

43 limit 42 to yr="1990 -Current" (205675)

44 exp Alcohol abuse/ (19451)

45 exp Alcohol Intoxication/ (10451)

46 exp Alcoholic Beverages/ (17080)

47 exp Drinking Behavior/ (33004)

48 (beer or wine$ or cider or alcopop$ or spirit or spirits).ti,ab. (25379)

49 alcohol$.ti,ab. (271310)

50 (drink$ adj2 (binge or excessive or harm$ or heavy or misus$ or abus$ or consum$)).ti,ab. (12748)

51 (intoxicat$ or inebriat$ or drunk$).ti,ab. (45337)

52 44 or 45 or 46 or 47 or 48 or 49 or 50 or 51 (348278)

53 (editorial or letter).pt. (1221865)

54 52 not 53 (339954)

55 limit 54 to yr="1990 -Current" (264679)

56 Unsafe Sex/ (1787)

57 multiple sexual partner$.ti,ab. (904)

58 multiple casual partner$.ti,ab. (10)

59 one time sex$ encounter$.ti,ab. (4)

60 one-time sex$ encounter$.ti,ab. (4)

61 (sex$ adj2 holiday$).ti,ab. (9)

62 casual sex$.ti,ab. (562)

63 casual partner$.ti,ab. (759)

64 non-regular sex$ partner$.ti,ab. (25)

65 non regular sex$ partner$.ti,ab. (25)

66 (unprotected adj2 intercourse).ti,ab. (2026)

67 (unprotected adj2 sex$).ti,ab. (2874)

68 (condomless adj (sex$ or intercourse)).ti,ab. (7)

69 (condom free adj (sex$ or intercourse)).ti,ab. (2)

70 (RUAI or UAI).ti,ab. (334)

71 (barebacking or bareback sex$ or bugchas$ or bug chas$).ti,ab. (76)

72 anal intercourse.ti,ab. (1706)

73 anal sex.ti,ab. (1248)

74 or/56-73 (8581)

75 sexual behavior/ (76516)

76 high risk behavior/ (12921)

77 75 and 76 (3654)

78 (risk$ sex$ behavio$ or unsafe sex$).ti,ab. (3475)

79 74 or 77 or 78 (13138)

80 (comment or editorial).pt. (419336)

81 79 not 80 (13018)

82 limit 81 to yr="1990 -Current" (12731)

83 exp drug abuse/ (55305)

84 substance abuse/ (35403)

85 ((drug$ or substance$) adj2 (use$ or using or abuse$ or abusing or misuse$ or misusing)).ti,ab. (164609)

86 ((heroin or opiate$ or cocaine or crack) adj2 (use$ or using or abuse$ or abusing or misuse$ or misusing)).ti,ab. (14761)

87 ((cannabis or marijuana) adj2 (use$ or using or abuse$ or abusing or misuse$ or misusing)).ti,ab. (7674)

88 ((benzodiazepine$ or amphetamine$ or methamphetamine$ or MDMA or ecstasy) adj2 (use$ or using or abuse$ or abusing or misuse$ or misusing)).ti,ab. (7625)

89 (solvent$ adj2 (use$ or using or abuse$ or abusing or misuse$ or misusing)).ti,ab. (7981)

90 street drug$.ti,ab. (534)

91 (prescri$ drug$ adj2 (use$ or using or abuse$ or abusing or misuse$ or misusing)).ti,ab. (1575)

92 polydrug use$.ti,ab. (647)

93 inject$ drug use$.ti,ab. (8306)

94 (needle$ adj3 (share$ or sharing)).ti,ab. (1357)

95 (syringe$ adj3 (share$ or sharing)).ti,ab. (545)

96 or/83-95 (225114)

97 (editorial or comment).pt. (419336)

98 96 not 97 (221774)

99 limit 98 to yr="1990 -Current" (195517)

100 sunbathing/ (232)

101 sunscreen/ or sunburn/ (8806)

102 (sunbath$ or sunscreen$ or sunburn$ or suntan$ or sunbed$).ti,ab. (6729)

103 (sun bath$ or sun screen$ or sun burn$ or sun tan$ or sun bed$).ti,ab. (366)

104 sun protect$.ti,ab. (2395)

105 (tanning adj (bed$ or salon$ or studio$)).ti,ab. (236)

106 100 or 101 or 102 or 103 or 104 or 105 (11950)

107 (comment or editorial).pt. (419336)

108 106 not 107 (11733)

109 limit 108 to yr="1990 -Current" (10424)

110 dental health/ or dental caries/ (38716)

111 mouth hygiene/ or tooth brushing/ (22100)

112 (dental care or dental health or dental hygiene).ti,ab. (15599)

113 (oral care or oral health or oral hygiene).ti,ab. (19567)

114 (gingival care or gingival health or gingival hygiene).ti,ab. (670)

115 ((unsupervised or irregular$ or regular$ or lack or seldom or never or infrequent$ or frequen$ or insufficient$) adj2 (toothbrushing or flossing)).ti,ab. (326)

116 ((irregular$ or regular$ or seldom or lack or never or infrequent$ or frequen$) adj3 (dental or dentist$)).ti,ab. (2593)

117 ((irregular$ or regular$ or seldom or lack or never or infrequent$ or frequen$) adj3 dental visit$).ti,ab. (279)

118 ((irregular$ or regular$ or seldom or lack or never or infrequent$ or frequen$) adj3 dental attendance).ti,ab. (84)

119 (clean$ teeth adj2 (irregular$ or regular$ or infrequent$ or frequen$ or seldom or never)).ti,ab. (3)

120 (brush$ teeth adj2 (irregular$ or regular$ or infrequent$ or frequen$ or seldom or never)).ti,ab. (12)

121 ((sweet$ drink$ or fizzy drink$ or sugary snack$ or sweets or confectionery) adj6 (tooth or teeth or dental or oral or caries or decay)).ti,ab. (161)

122 or/110-121 (76024)

123 (editorial or comment).pt. (419336)

124 122 not 123 (75176)

125 limit 124 to yr="1990 -Current" (45742)

126 patient compliance/ (88311)

127 treatment refusal/ (10800)

128 126 and 127 (1176)

129 (non-adherence adj2 (patient$ or medication$ or screen$ or treatment or therapy or immunisation or immunization or regimen$ or drug$)).ti,ab. (1050)

130 (nonadherence adj2 (patient$ or medication$ or screen$ or treatment or therapy or immunisation or immunization or regimen$ or drug$)).ti,ab. (1176)

131 (low adherence adj2 (patient$ or medication$ or screen$ or treatment or therapy or immunisation or immunization or regimen$ or drug$)).ti,ab. (128)

132 (poor adherence adj2 (patient$ or medication$ or screen$ or treatment or therapy or immunisation or immunization or regimen$ or drug$)).ti,ab. (581)

133 ((loss or lack or failure or barrier$ or impediment$ or selective or minimal) adj2 adherence).ti,ab. (1601)

134 (non-compliance adj2 (patient$ or medication$ or screen$ or treatment or therapy or immunisation or immunization or regimen$ or drug$)).ti,ab. (1064)

135 (noncompliance adj2 (patient$ or medication$ or screen$ or treatment or therapy or immunisation or immunization or regimen$ or drug$)).ti,ab. (1437)

136 (low compliance adj2 (patient$ or medication$ or screen$ or treatment or therapy or immunisation or immunization or regimen$ or drug$)).ti,ab. (134)

137 (poor compliance adj2 (patient$ or medication$ or screen$ or treatment or therapy or immunisation or immunization or regimen$ or drug$)).ti,ab. (630)

138 ((loss or lack or failure or barrier$ or impediment$ or selective or minimal) adj2 compliance).ti,ab. (1504)

139 treatment refusal/ (10800)

140 mass screening/ (46649)

141 139 and 140 (60)

142 (non-attend$ adj3 screen$).ti,ab. (67)

143 (nonattend$ adj3 screen$).ti,ab. (20)

144 (non-attend$ adj3 appoint$).ti,ab. (73)

145 (nonattend$ adj3 appoint$).ti,ab. (27)

146 (non-attend$ adj3 (check-up$ or checkup$)).ti,ab. (3)

147 (nonattend$ adj3 (check-up$ or checkup$)).ti,ab. (0)

148 (non-attend$ adj3 (mammogra$ or smear test$ or PAP test$ or breast exam$ or CBE)).ti,ab. (15)

149 (nonattend$ adj3 (mammogra$ or smear test$ or PAP test$ or breast exam$ or CBE)).ti,ab. (5)

150 128 or 129 or 130 or 131 or 132 or 133 or 134 or 135 or 136 or 137 or 138 or 141 or 142 or 143 or 144 or 145 or 146 or 147 or 148 or 149 (10157)

151 (editorial or comment).pt. (419336)

152 150 not 151 (10098)

153 limit 152 to yr="1990 -Current" (9496)

154 seatbelt/ (3537)

155 (seat belt$ or seatbelt$).ti,ab. (2936)

156 seat restraint$.ti,ab. (44)

157 passenger$ restraint$.ti,ab. (58)

158 driver$ restraint$.ti,ab. (12)

159 ((unbelted or unrestrained) adj2 (driver$ or passenger$)).ti,ab. (113)

160 154 or 155 or 156 or 157 or 158 or 159 (4781)

161 helmet/ (3163)

162 (cycle helmet$ or bike helmet$ or bicycle helmet$).ti,ab. (554)

163 161 or 162 (3239)

164 protective equipment/ (8912)

165 fire/ or smoke/ (14286)

166 164 and 165 (133)

167 (smoke adj (alarm$ or sensor$)).ti,ab. (163)

168 (fire adj (alarm$ or sensor$)).ti,ab. (64)

169 166 or 167 or 168 (328)

170 drunken driving/ (1767)

171 (drink$ adj2 (drive$ or driving)).ti,ab. (1490)

172 alcohol impaired driv$.ti,ab. (227)

173 170 or 171 or 172 (2911)

174 160 or 163 or 169 or 173 (10793)

175 (letter or comment).pt. (802529)

176 174 not 175 (10247)

177 limit 176 to yr="1990 -Current" (8410)

178 pathological gambling/ (4176)

179 (gambling or gambler).mp. or gamblers.ti,ab. [mp=title, abstract, subject headings, heading word, drug trade name, original title, device manufacturer, drug manufacturer, device trade name, keyword] (5221)

180 178 or 179 (5221)

181 (editorial or comment).pt. (419336)

182 180 not 181 (5091)

183 limit 182 to yr="1990 -Current" (4713)

184 (18 and 35) or (18 and 43) or (18 and 55) or (18 and 82) or (18 and 99) or (18 and 109) or (18 and 125) or (18 and 153) or (18 and 177) or (18 and 183) (65809)

185 (35 and 18) or (35 and 43) or (35 and 55) or (35 and 82) or (35 and 99) or (35 and 109) or (35 and 125) or (35 and 153) or (35 and 177) or (35 and 183) (65478)

186 (43 and 18) or (43 and 35) or (43 and 55) or (43 and 82) or (43 and 99) or (43 and 109) or (43 and 125) or (43 and 153) or (43 and 177) or (43 and 183) (63197)

187 (55 and 18) or (55 and 35) or (55 and 43) or (55 and 82) or (55 and 99) or (55 and 109) or (55 and 125) or (55 and 153) or (55 and 177) or (55 and 183) (74420)

188 (82 and 18) or (82 and 35) or (82 and 43) or (82 and 55) or (82 and 99) or (82 and 109) or (82 and 125) or (82 and 153) or (82 and 177) or (82 and 183) (4969)

189 (99 and 18) or (99 and 35) or (99 and 43) or (99 and 55) or (99 and 82) or (99 and 109) or (99 and 125) or (99 and 153) or (99 and 177) or (99 and 183) (46794)

190 (109 and 18) or (109 and 35) or (109 and 43) or (109 and 55) or (109 and 82) or (109 and 99) or (109 and 125) or (109 and 153) or (109 and 177) or (109 and 183) (1171)

191 (125 and 18) or (125 and 35) or (125 and 43) or (125 and 55) or (125 and 82) or (125 and 99) or (125 and 109) or (125 and 153) or (125 and 177) or (125 and 183) (4714)

192 (153 and 18) or (153 and 35) or (153 and 43) or (153 and 55) or (153 and 82) or (153 and 99) or (153 and 109) or (153 and 125) or (153 and 177) or (153 and 183) (1777)

193 (177 and 18) or (177 and 35) or (177 and 43) or (177 and 55) or (177 and 82) or (177 and 99) or (177 and 109) or (177 and 125) or (177 and 153) or (177 and 183) (3498)

194 (183 and 18) or (183 and 35) or (183 and 43) or (183 and 55) or (183 and 82) or (183 and 99) or (183 and 109) or (183 and 125) or (183 and 153) or (183 and 177) (1665)

195 184 or 185 or 186 or 187 or 188 or 189 or 190 or 191 or 192 or 193 or 194 (151818)

196 animal experiment/ (1555828)

197 195 not 196 (148854)

198 random$.tw. (776012)

199 placebo$.mp. (292102)

200 double-blind$.tw. (133299)

201 198 or 199 or 200 (962134)

202 197 and 201 (18010)

203 trial.ti. (139456)

204 (evaluate$ or evaluation or evaluating).ti. (425668)

205 evaluation study.ab. (1625)

206 (intervention or program or programme).ti. (152665)

207 interrupted time series.ti,ab. (926)

208 (before-after adj2 study).ti,ab. (574)

209 (before adj3 study).ti,ab. (11759)

210 experimental study.ti,ab. (40898)

211 quasi-experimental study.ti,ab. (1302)

212 (pre post or pre-post).ti,ab. (5371)

213 203 or 204 or 205 or 206 or 207 or 208 or 209 or 210 or 211 or 212 (752824)

214 197 and 213 (8912)

215 202 or 214 (23682)

216 community.ti,ab. (298184)

217 community-based.ti,ab. (36552)

218 (neighbourhood$ or neighborhood$).ti,ab. (15933)

219 urban communities.ti,ab. (1067)

220 rural communities.ti,ab. (4091)

221 Rural population/ (27506)

222 urban population/ (32759)

223 ((disadvantaged or poor or deprived) adj communities).ti,ab. (775)

224 (work or worksite or workplace).ti,ab. (629998)

225 work/ or work environment/ (37858)

226 (web-based or web or website or online or internet or computer or computer-tailored or computer-based or online or email or telephone).ti,ab. (316028)

227 exp mass communication/ (330251)

228 ((parent$ or family or women$ or woman$ or sure start) adj2 (centre$ or center$ or co-op or cooperative or clinic$)).ti,ab. (17334)

229 health center/ (19307)

230 (GP practice$ or general practice or family practice or primary care).ti,ab. (111088)

231 General Practice/ (66041)

232 Family Practice/ (66041)

233 ((emergency or outpatient) adj (department$ or clinic$ or ward$)).ti,ab. (81016)

234 (accident adj emergency).ti,ab. (269)

235 (campus$ or college$ or classroom$).ti,ab. (146443)

236 (church$ or home$ or home-based or pharmacy or pharmacies or night club$ or beer hall$).ti,ab. (384378)

237 216 or 217 or 218 or 219 or 220 or 221 or 222 or 223 or 224 or 225 or 226 or 227 or 228 or 229 or 230 or 231 or 232 or 233 or 234 or 235 or 236 (2124840)

238 215 and 237 (8092)

239 limit 238 to yr="1990 -Current" (8092)

240 (lifestyle adj2 (intervention$ or program$)).ti,ab. (4374)

241 (life style adj2 (intervention$ or program$)).ti,ab. (242)

242 (behavior$ change adj (intervention$ or program$)).ti,ab. (392)

243 (behaviour$ change adj (intervention$ or program$)).ti,ab. (222)

244 (multiple risk factor adj2 (program$ or intervention$)).ti,ab. (472)

245 (multifactorial lifestyle adj (intervention$ or program$)).ti,ab. (8)

246 (health behavior$ adj (program$ or intervention$)).ti,ab. (111)

247 (health behaviour$ adj (program$ or intervention$)).ti,ab. (31)

248 multiple health behavior$ change intervention$.ti,ab. (4)

249 multiple health behaviour$ change intervention$.ti,ab. (4)

250 multiple behavior$ risk factor$ intervention$.ti,ab. (1)

251 multiple behaviour$ risk factor$ intervention$.ti,ab. (0)

252 multiple behavior$ risk factor$ program$.ti,ab. (0)

253 multiple behaviour$ risk factor$ program$.ti,ab. (0)

254 multiple risk behaviour$ intervention$.ti,ab. (0)

255 multiple risk behavior$ intervention$.ti,ab. (4)

256 multiple risk behaviour$ program$.ti,ab. (0)

257 multiple risk behavior$ program$.ti,ab. (0)

258 240 or 241 or 242 or 243 or 244 or 245 or 246 or 247 or 248 or 249 or 250 or 251 or 252 or 253 or 254 or 255 or 256 or 257 (5775)

259 239 or 258 (13415)

260 limit 259 to yr="1990 -Current" (13176)

**MEDLINE**

**Via OvidSP**

**Search date 15^th^ January 2013**

**8279 records identified**

1 (healthy adj2 (diet$ or eating)).ti,ab. (4481)

2 (fruit$ adj2 (intake or consum$ or increase or portion$ or serving$ or frequenc$ or number$ or preference$ or choice$)).ti,ab. (4760)

3 (vegetable$ adj2 (intake or consum$ or increase or portion$ or serving$ or frequenc$ or number$ or preference$ or choice$)).ti,ab. (4803)

4 "5 a day".ti,ab. (133)

5 "five a day".ti,ab. (27)

6 ((food or diet$) adj (choice$ or frequenc$ or intake)).ti,ab. (48817)

7 Feeding Behavior/ (34992)

8 food habits/ or food preferences/ (25970)

9 nutrition therapy/ or exp diet therapy/ or exp diet/ (185426)

10 obesity/ or overweight/ (114303)

11 1 or 2 or 3 or 4 or 5 or 6 or 7 or 8 or 9 or 10 (349724)

12 (comment or editorial or letter).pt. (1206498)

13 11 not 12 (335271)

14 limit 13 to yr="1990 -Current" (229404)

15 (physical adj3 (fit$ or train$ or activ$ or endur$)).ti,ab. (59596)

16 (exercis$ adj3 (fit$ or train$ or activ$ or endur$)).ti,ab. (20958)

17 ((promot$ or uptak$ or encourag$ or increas$ or start$ or adher$) adj3 (exercis$ or gym$ or sport$ or fitness)).ti,ab. (20665)

18 ((decreas$ or reduc$ or discourag$) adj3 (sedentary or deskbound)).ti,ab. (424)

19 (sport$ or walk$ or running or jogging or bicycling or biking or swimming).ti,ab. (146110)

20 (active adj (travel$ or transport$ or commut$)).ti,ab. (6760)

21 physical fitness/ (20160)

22 exp Recreation/ (116348)

23 exp Exercise Therapy/ or exp exercise/ (118491)

24 running/ or jogging/ or swimming/ or walking/ (39259)

25 15 or 16 or 17 or 18 or 19 or 20 or 21 or 22 or 23 or 24 (341732)

26 (letter or editorial or comment).pt. (1206498)

27 25 not 26 (329528)

28 limit 27 to yr="1990 -Current" (262773)

29 exp smoking/ (111314)

30 (smoking or antismoking or anti-smoking).ti,ab. (133027)

31 (smoker or smokers).ti,ab. (52919)

32 tobacco/ or tobacco.ti,ab. (67958)

33 29 or 30 or 31 or 32 (224900)

34 (letter or editorial or comment).pt. (1206498)

35 33 not 34 (215145)

36 limit 35 to yr="1990 -Current" (172420)

37 exp Alcohol Drinking/ (46742)

38 exp Alcoholic Intoxication/ (10467)

39 exp Alcoholic Beverages/ (12721)

40 exp Drinking Behavior/ (51989)

41 (beer or wine$ or cider or alcopop$ or spirit or spirits).ti,ab. (20472)

42 alcohol$.ti,ab. (212046)

43 (drink$ adj2 (binge or excessive or harm$ or heavy or misus$ or abus$ or consum$)).ti,ab. (9976)

44 (intoxicat$ or inebriat$ or drunk$).ti,ab. (37775)

45 37 or 38 or 39 or 40 or 41 or 42 or 43 or 44 (279044)

46 (comment or editorial or letter).pt. (1206498)

47 45 not 46 (271011)

48 limit 47 to yr="1990 -Current" (191560)

49 Unsafe Sex/ (2229)

50 multiple sexual partner$.ti,ab. (774)

51 multiple casual partner$.ti,ab. (10)

52 one time sex$ encounter$.ti,ab. (3)

53 one-time sex$ encounter$.ti,ab. (3)

54 (sex$ adj2 holiday$).ti,ab. (8)

55 casual sex$.ti,ab. (500)

56 casual partner$.ti,ab. (665)

57 non-regular sex$ partner$.ti,ab. (22)

58 non regular sex$ partner$.ti,ab. (22)

59 (unprotected adj2 intercourse).ti,ab. (1910)

60 (unprotected adj2 sex$).ti,ab. (2595)

61 (condomless adj (sex$ or intercourse)).ti,ab. (10)

62 (condom free adj (sex$ or intercourse)).ti,ab. (2)

63 (RUAI or UAI).ti,ab. (288)

64 (barebacking or bareback sex$ or bugchas$ or bug chas$).ti,ab. (68)

65 anal intercourse.ti,ab. (1539)

66 anal sex.ti,ab. (1074)

67 or/49-66 (7982)

68 sexual behavior/ (38258)

69 risk taking/ (17004)

70 68 and 69 (4893)

71 (risk$ sex$ behavio$ or unsafe sex$).ti,ab. (2939)

72 67 or 70 or 71 (12820)

73 (letter or comment or editorial).pt. (1206498)

74 72 not 73 (12474)

75 limit 74 to yr="1990 -Current" (12101)

76 substance-related disorders/ or inhalant abuse/ or marijuana abuse/ or substance abuse, intravenous/ (86397)

77 Drug Users/ (838)

78 ((drug$ or substance$) adj2 (use$ or using or abuse$ or abusing or misuse$ or misusing)).ti,ab. (128121)

79 ((heroin or opiate$ or cocaine or crack) adj2 (use$ or using or abuse$ or abusing or misuse$ or misusing)).ti,ab. (12026)

80 ((cannabis or marijuana) adj2 (use$ or using or abuse$ or abusing or misuse$ or misusing)).ti,ab. (6159)

81 ((benzodiazepine$ or amphetamine$ or methamphetamine$ or MDMA or ecstasy) adj2 (use$ or using or abuse$ or abusing or misuse$ or misuising)).ti,ab. (5991)

82 (solvent$ adj2 (use$ or using or abuse$ or abusing or misuse$ or misusing)).ti,ab. (5945)

83 street drug$.ti,ab. (423)

84 (prescri$ drug$ adj2 (use$ or using or abuse$ or abusing or misuse$ or misusing)).ti,ab. (1222)

85 polydrug use$.ti,ab. (568)

86 inject$ drug use$.ti,ab. (7317)

87 (needle adj3 shar$).ti,ab. (981)

88 (syringe$ adj3 shar$).ti,ab. (490)

89 or/76-88 (189083)

90 (letter or editorial or comment).pt. (1206498)

91 89 not 90 (180981)

92 limit 91 to yr="1990 -Current" (142389)

93 sunbathing/ (238)

94 sunscreening agents/ (3593)

95 (sunbath$ or sunscreen$ or sunburn$ or suntan$ or sunbed$).ti,ab. (5167)

96 (sun bath$ or sun screen$ or sun burn$ or sun tan$ or sun bed$).ti,ab. (260)

97 (sun protect$ or sun bed$ or sun tan$).ti,ab. (1912)

98 (tanning adj (bed$ or salon$ or studio$)).ti,ab. (187)

99 93 or 94 or 95 or 96 or 97 or 98 (7575)

100 (letter or comment or editorial).pt. (1206498)

101 99 not 100 (7068)

102 limit 101 to yr="1990 -Current" (6058)

103 (poor dental care or poor dental health or poor dental hygiene).ti,ab. (148)

104 (poor oral care or poor oral health or poor oral hygiene).ti,ab. (1070)

105 (poor gingival care or poor gingival health or poor gingival hygiene).ti,ab. (4)

106 ((unsupervised or irregular$ or lack or never or infrequent$ or insufficient$) adj2 (toothbrushing or flossing)).ti,ab. (32)

107 ((irregular$ or lack or never or infrequent$) adj3 (dental or dentist$)).ti,ab. (566)

108 ((irregular$ or lack or never or infrequent$) adj3 dental visit$).ti,ab. (35)

109 ((irregular$ or lack or never or infrequent$) adj3 dental attendance).ti,ab. (21)

110 (cleaning teeth adj2 (irregular$ or infrequent$ or never)).ti,ab. (0)

111 (brushing teeth adj2 (irregular$ or infrequent$ or never)).ti,ab. (2)

112 ((sweet$ drink$ or fizzy drink$ or sugary snack$ or sweets or confectionery) adj6 (tooth or teeth or dental or oral or caries or decay)).ti,ab. (152)

113 or/103-112 (1904)

114 (letter or editorial or comment).pt. (1206498)

115 113 not 114 (1885)

116 limit 115 to yr="1990 -Current" (1669)

117 patient compliance/ (43324)

118 treatment refusal/ (10087)

119 117 and 118 (798)

120 (non-adherence adj2 (patient$ or medication$ or screen$ or treatment or therapy or immunisation or regimen$ or drug$)).ti,ab. (609)

121 (nonadherence adj2 (patient$ or medication$ or screen$ or treatment or therapy or immunisation or regimen$ or drug$)).ti,ab. (862)

122 (low adherence adj2 (patient$ or medication$ or screen$ or treatment or therapy or immunisation or regimen$ or drug$)).ti,ab. (65)

123 (poor adherence adj2 (patient$ or medication$ or screen$ or treatment or therapy or immunisation or regimen$ or drug$)).ti,ab. (372)

124 ((loss or lack or failure or barrier$ or impediment$ or selective or minimal) adj2 adherence).ti,ab. (1133)

125 (non-compliance adj2 (patient$ or medication$ or screen$ or treatment or therapy or immunisation or regimen$ or drug$)).ti,ab. (710)

126 (noncompliance adj2 (patient$ or medication$ or screen$ or treatment or therapy or immunisation or regimen$ or drug$)).ti,ab. (1119)

127 (low compliance adj2 (patient$ or medication$ or screen$ or treatment or therapy or immunisation or regimen$ or drug$)).ti,ab. (90)

128 (poor compliance adj2 (patient$ or medication$ or screen$ or treatment or therapy or immunisation or regimen$ or drug$)).ti,ab. (419)

129 ((loss or lack or failure or barrier$ or impediment$ or selective or minimal) adj2 compliance).ti,ab. (1074)

130 treatment refusal/ (10087)

131 mass screening/ (75615)

132 130 and 131 (131)

133 (non-attend$ adj3 screen$).ti,ab. (52)

134 (nonattend$ adj3 screen$).ti,ab. (18)

135 (non-attend$ adj3 appoint$).ti,ab. (39)

136 (nonattend$ adj3 appoint$).ti,ab. (19)

137 (non-attend$ adj3 mammograph$).ti,ab. (10)

138 (nonattend$ adj3 mammograph$).ti,ab. (5)

139 119 or 120 or 121 or 122 or 123 or 124 or 125 or 126 or 127 or 128 or 129 or 132 or 133 or 134 or 135 or 136 or 137 or 138 (7135)

140 (letter or editorial or comment).pt. (1206498)

141 139 not 140 (6985)

142 limit 141 to yr="1990 -Current" (6424)

143 seat belts/ (3155)

144 seat belts.ti,ab. (938)

145 seat restraints.ti,ab. (22)

146 passenger$ restraint$.ti,ab. (55)

147 driver$ restraint$.ti,ab. (12)

148 ((unbelted or unrestrained) adj2 (driver$ or passenger$)).ti,ab. (108)

149 143 or 144 or 145 or 146 or 147 or 148 (3551)

150 head protective devices/ (2271)

151 (cycle helmet$ or bike helmet$ or bicycle helmet$).ti,ab. (508)

152 150 or 151 (2371)

153 protective devices/ (5590)

154 fires/ or smoke/ (11457)

155 153 and 154 (160)

156 (smoke adj (alarm$ or sensor$)).ti,ab. (141)

157 (fire adj (alarm$ or sensor$)).ti,ab. (49)

158 155 or 156 or 157 (296)

159 automobile driving/ (12384)

160 alcoholic intoxication/ or alcohol drinking/ (54827)

161 159 and 160 (2350)

162 (drink$ adj2 (drive$ or driving)).ti,ab. (1211)

163 alcohol impaired driv$.ti,ab. (201)

164 161 or 162 or 163 (2970)

165 149 or 152 or 158 or 164 (8907)

166 (editorial or letter or comment).pt. (1206498)

167 165 not 166 (8121)

168 limit 167 to yr="1990 -Current" (5884)

169 Gambling/ (2929)

170 gambling.ti,ab. (3270)

171 169 or 170 (3950)

172 (letter or editorial or comment).pt. (1206498)

173 171 not 172 (3727)

174 limit 173 to yr="1990 -Current" (3360)

175 (14 and 28) or (14 and 36) or (14 and 48) or (14 and 75) or (14 and 92) or (14 and 102) or (14 and 116) or (14 and 142) or (14 and 168) or (14 and 174) (43181)

176 (28 and 14) or (28 and 36) or (28 and 48) or (28 and 75) or (28 and 92) or (28 and 102) or (28 and 116) or (28 and 142) or (28 and 168) or (28 and 174) (40213)

177 (36 and 14) or (36 and 28) or (36 and 48) or (36 and 75) or (36 and 92) or (36 and 102) or (36 and 116) or (36 and 142) or (36 and 168) or (36 and 174) (47465)

178 (48 and 14) or (48 and 28) or (48 and 36) or (48 and 75) or (48 and 92) or (48 and 102) or (48 and 116) or (48 and 142) or (48 and 168) or (48 and 174) (58712)

179 (75 and 14) or (75 and 28) or (75 and 36) or (75 and 48) or (75 and 92) or (75 and 102) or (75 and 116) or (75 and 142) or (75 and 168) or (75 and 174) (4991)

180 (92 and 14) or (92 and 28) or (92 and 36) or (92 and 48) or (92 and 75) or (92 and 102) or (92 and 116) or (92 and 142) or (92 and 168) or (92 and 174) (34593)

181 (102 and 14) or (102 and 28) or (102 and 36) or (102 and 48) or (102 and 75) or (102 and 92) or (102 and 116) or (102 and 142) or (102 and 168) or (102 and 174) (748)

182 (116 and 14) or (116 and 28) or (116 and 36) or (116 and 48) or (116 and 75) or (116 and 92) or (116 and 102) or (116 and 142) or (116 and 168) or (116 and 174) (427)

183 (142 and 14) or (142 and 28) or (142 and 36) or (142 and 48) or (142 and 75) or (142 and 92) or (142 and 102) or (142 and 116) or (142 and 168) or (142 and 174) (1119)

184 (168 and 14) or (168 and 28) or (168 and 36) or (168 and 48) or (168 and 75) or (168 and 92) or (168 and 102) or (168 and 116) or (168 and 142) or (168 and 174) (3157)

185 (174 and 14) or (174 and 28) or (174 and 36) or (174 and 48) or (174 and 75) or (174 and 92) or (174 and 102) or (174 and 116) or (174 and 142) or (174 and 168) (1152)

186 175 or 176 or 177 or 178 or 179 or 180 or 181 or 182 or 183 or 184 or 185 (107085)

187 randomized controlled trial.pt. (337493)

188 controlled clinical trial.pt. (84936)

189 randomized.ab. (254740)

190 placebo.ab. (139525)

191 clinical trials as topic.sh. (161725)

192 randomly.ab. (186500)

193 trial.ti. (108691)

194 187 or 188 or 189 or 190 or 191 or 192 or 193 (811148)

195 exp animals/ not humans.sh. (3744372)

196 194 not 195 (749287)

197 186 and 196 (11145)

198 trial.ti. (108691)

199 (evaluate$ or evaluation or evaluating).ti. (347968)

200 evaluation study.ab. (1233)

201 (intervention or program or programme).ti. (126411)

202 interrupted time series.ti,ab. (775)

203 (before-after adj2 study).ti,ab. (433)

204 (before adj3 study).ti,ab. (8983)

205 experimental study.ti,ab. (36557)

206 quasi-experimental study.ti,ab. (1099)

207 quasi experimental study.ti,ab. (1099)

208 (pre post or pre-post).ti,ab. (3514)

209 198 or 199 or 200 or 201 or 202 or 203 or 204 or 205 or 206 or 207 or 208 (613434)

210 186 and 209 (6337)

211 197 or 210 (14689)

212 community.ti,ab. (251569)

213 community-based.ti,ab. (30732)

214 (neighbourhood$ or neighborhood$).ti,ab. (14895)

215 urban communities.ti,ab. (950)

216 rural communities.ti,ab. (3664)

217 Residence Characteristics/ (17196)

218 ((disadvantaged or poor or deprived) adj communities).ti,ab. (681)

219 Poverty Areas/ (3826)

220 (work or worksite or workplace).ti,ab. (521324)

221 Workplace/ or Employment/ (45082)

222 (web-based or web or website or online or internet or computer or computer-tailored or computer-based or online or email or telephone).ti,ab. (262762)

223 Internet/ or Telephone/ or Electronic Mail/ (51839)

224 ((parent$ or family or women$ or woman$ or sure start) adj2 (centre$ or center$ or co-op or cooperative or clinic$)).ti,ab. (14814)

225 Community Health Centers/ (5572)

226 (GP practice$ or general practice or family practice or primary care).ti,ab. (91024)

227 General Practice/ or Family Practice/ (60749)

228 ((emergency or outpatient) adj (department$ or clinic$ or ward$)).ti,ab. (61137)

229 (accident adj emergency).ti,ab. (232)

230 (campus$ or college$ or classroom$).ti,ab. (88030)

231 (church$ or home$ or home-based or pharmacy or pharmacies or night club$ or beer hall$).ti,ab. (303096)

232 212 or 213 or 214 or 215 or 216 or 217 or 218 or 219 or 220 or 221 or 222 or 223 or 224 or 225 or 226 or 227 or 228 or 229 or 230 or 231 (1540701)

233 211 and 232 (4641)

234 (lifestyle adj2 (intervention$ or program$)).ti,ab. (3019)

235 (life style adj2 (intervention$ or program$)).ti,ab. (150)

236 (behavior$ change adj (intervention$ or program$)).ti,ab. (350)

237 (behaviour$ change adj (intervention$ or program$)).ti,ab. (175)

238 (multiple risk factor adj2 (program$ or intervention$)).ti,ab. (404)

239 (multifactorial lifestyle adj (intervention$ or program$)).ti,ab. (6)

240 (health behavior$ adj (program$ or intervention$)).ti,ab. (96)

241 (health behaviour$ adj (program$ or intervention$)).ti,ab. (23)

242 multiple health behavior$ change intervention$.ti,ab. (4)

243 multiple health behaviour$ change intervention$.ti,ab. (3)

244 multiple behavior$ risk factor$ intervention$.ti,ab. (1)

245 multiple behaviour$ risk factor$ intervention$.ti,ab. (0)

246 multiple behavior$ risk factor$ program$.ti,ab. (0)

247 multiple behaviour$ risk factor$ program$.ti,ab. (0)

248 multiple risk behaviour$ intervention$.ti,ab. (0)

249 multiple risk behavior$ intervention$.ti,ab. (2)

250 multiple risk behaviour$ program$.ti,ab. (0)

251 multiple risk behavior$ program$.ti,ab. (0)

252 234 or 235 or 236 or 237 or 238 or 239 or 240 or 241 or 242 or 243 or 244 or 245 or 246 or 247 or 248 or 249 or 250 or 251 (4171)

253 233 or 252 (8504)

254 limit 253 to yr="1990 -Current" (8279)

**PsycINFO**

**Via OVIDSP**

**Search date 15^th^ January 2013**

**5475 records identified**

1 (health$ adj2 (diet$ or eating or food or foods)).ti,ab. (3071)

2 (unhealth$ adj2 (diet$ or eating or food or foods)).ti,ab. (668)

3 (fruit$ adj2 (eat or eats or eating or intake or consum$ or increas$ or portion$ or serving$ or frequenc$ or number$ or preference$ or choice$)).ti,ab. (1184)

4 (vegetable$ adj2 (eat or eats or eating or intake or consum$ or increas$ or portion$ or serving$ or frequenc$ or number$ or preference$ or choice$)).ti,ab. (1110)

5 "5 a day".ti,ab. (65)

6 "five a day".ti,ab. (7)

7 (junk food or fast food).ti,ab. (717)

8 ((decreas$ or reduc$ or discourag$ or limit$ or lessen or eat$ less) adj2 (salt or fat)).ti,ab. (601)

9 (food adj (choice$ or frequenc$ or select$)).ti,ab. (1881)

10 Eating Behavior/ (5722)

11 food preferences/ (2341)

12 nutrition/ or diets/ (11048)

13 obesity/ or overweight/ (12121)

14 1 or 2 or 3 or 4 or 5 or 6 or 7 or 8 or 9 or 10 or 11 or 12 or 13 (30062)

15 (comment reply or editorial or letter).dt. (111964)

16 14 not 15 (28907)

17 limit 16 to yr="1990 -Current" (27666)

18 (physical$ adj3 (fit$ or train$ or activ$ or inactiv$ or endur$)).ti,ab. (17910)

19 (exercis$ adj3 (fit$ or train$ or activ$ or endur$)).ti,ab. (3182)

20 ((promot$ or uptak$ or encourag$ or increas$ or start$ or adher$) adj3 (exercis$ or gym$ or sport$ or fitness)).ti,ab. (3250)

21 ((decreas$ or reduc$ or discourag$) adj3 (sedentary or deskbound)).ti,ab. (138)

22 (sedentary behaviour$ or sedentary behavior$ or sedentary lifestyle$ or sedentariness).ti,ab. (972)

23 ((watch$ or view$) adj2 (tv or television)).ti,ab. (2645)

24 (sport$ or walk$ or running or jogging or bicycling or biking or swimming).ti,ab. (37788)

25 (active adj (travel$ or transport$ or commut$)).ti,ab. (247)

26 physical fitness/ (2316)

27 exp Recreation/ or leisure time/ (21261)

28 Exercise/ (11647)

29 running/ or swimming/ or walking/ (4547)

30 18 or 19 or 20 or 21 or 22 or 23 or 24 or 25 or 26 or 27 or 28 or 29 (78235)

31 (letter or editorial or comment reply).dt. (111964)

32 30 not 31 (75686)

33 limit 32 to yr="1990 -Current" (72119)

34 exp tobacco smoking/ (18254)

35 (smoking or antismoking or anti-smoking).ti,ab. (26507)

36 (smoker or smokers).ti,ab. (12049)

37 tobacco.ti,ab. (11640)

38 34 or 35 or 36 or 37 (33838)

39 (letter or editorial or comment reply).dt. (111964)

40 38 not 39 (32291)

41 limit 40 to yr="1990 -Current" (31091)

42 exp Alcohol abuse/ (28184)

43 exp Alcohol Intoxication/ (1644)

44 exp alcoholism/ (16531)

45 exp alcohol drinking patterns/ (39297)

46 (beer or wine$ or cider or alcopop$ or spirit or spirits).ti,ab. (8183)

47 alcohol$.ti,ab. (70761)

48 (drink$ adj2 (binge or excessive or harm$ or heavy or misus$ or abus$ or consum$)).ti,ab. (6515)

49 (intoxicat$ or inebriat$ or drunk$).ti,ab. (6330)

50 42 or 43 or 44 or 45 or 46 or 47 or 48 or 49 (83220)

51 (comment reply or editorial or letter).dt. (111964)

52 50 not 51 (79882)

53 limit 52 to yr="1990 -Current" (74048)

54 sexual risk taking/ (5405)

55 multiple sexual partner$.ti,ab. (334)

56 multiple casual partner$.ti,ab. (4)

57 one time sex$ encounter$.ti,ab. (5)

58 one-time sex$ encounter$.ti,ab. (5)

59 (sex$ adj2 holiday$).ti,ab. (4)

60 casual sex$.ti,ab. (438)

61 casual partner$.ti,ab. (423)

62 non-regular sex$ partner$.ti,ab. (9)

63 non regular sex$ partner$.ti,ab. (9)

64 (unprotected adj2 intercourse).ti,ab. (861)

65 (unprotected adj2 sex$).ti,ab. (1647)

66 (condomless adj (sex$ or intercourse)).ti,ab. (9)

67 (condom free adj (sex$ or intercourse)).ti,ab. (0)

68 (RUAI or UAI).ti,ab. (211)

69 (barebacking or bareback sex$ or bugchas$ or bug chas$).ti,ab. (99)

70 anal intercourse.ti,ab. (881)

71 anal sex.ti,ab. (700)

72 or/54-71 (7568)

73 (risk$ sex$ behavio$ or unsafe sex$).ti,ab. (2595)

74 72 or 73 (8613)

75 (letter or comment reply or editorial).dt. (111964)

76 74 not 75 (8354)

77 limit 76 to yr="1990 -Current" (8300)

78 exp drug abuse/ (66954)

79 exp drug dependency/ (16114)

80 ((drug$ or substance$) adj2 (use$ or using or abuse$ or abusing or misuse$ or misusing)).ti,ab. (69183)

81 ((heroin or opiate$ or cocaine or crack) adj2 (use$ or using or abuse$ or abusing or misuse$ or misusing)).ti,ab. (7270)

82 ((cannabis or marijuana) adj2 (use$ or using or abuse$ or abusing or misuse$ or misusing)).ti,ab. (5254)

83 ((benzodiazepine$ or amphetamine$ or methamphetamine$ or MDMA or ecstasy) adj2 (use$ or using or abuse$ or abusing or misuse$ or misusing)).ti,ab. (3569)

84 (solvent$ adj2 (use$ or using or abuse$ or abusing or misuse$ or misusing)).ti,ab. (178)

85 street drug$.ti,ab. (210)

86 (prescri$ drug$ adj2 (use$ or using or abuse$ or abusing or misuse$ or misusing)).ti,ab. (571)

87 polydrug use$.ti,ab. (543)

88 inject$ drug use$.ti,ab. (3215)

89 (needle$ adj3 (share$ or sharing)).ti,ab. (702)

90 (syringe$ adj3 (share$ or sharing)).ti,ab. (241)

91 or/78-90 (108592)

92 (letter or editorial or comment reply).dt. (111964)

93 91 not 92 (103820)

94 limit 93 to yr="1990 -Current" (97759)

95 (sunbath$ or sunscreen$ or sunburn$ or suntan$ or sunbed$).ti,ab. (284)

96 (sun bath$ or sun screen$ or sun burn$ or sun tan$ or sun bed$).ti,ab. (24)

97 sun protect$.ti,ab. (245)

98 (tanning adj (bed$ or salon$ or studio$)).ti,ab. (23)

99 95 or 96 or 97 or 98 (427)

100 (letter or comment reply or editorial).dt. (111964)

101 99 not 100 (420)

102 limit 101 to yr="1990 -Current" (418)

103 dental health/ (168)

104 oral health/ (369)

105 (dental care or dental health or dental hygiene).ti,ab. (624)

106 (oral care or oral health or oral hygiene).ti,ab. (684)

107 (gingival care or gingival health or gingival hygiene).ti,ab. (1)

108 ((unsupervised or irregular$ or regular$ or seldom or lack or never or infrequent$ or frequen$ or insufficient$) adj2 (toothbrushing or flossing)).ti,ab. (19)

109 ((irregular$ or regular$ or seldom or lack or never or infrequent$ or frequen$) adj3 (dental or dentist$)).ti,ab. (102)

110 ((irregular$ or regular$ or seldom or lack or never or infrequent$ or frequen$) adj3 dental visit$).ti,ab. (13)

111 ((irregular$ or regular$ or seldom or lack or never or infrequent$ or frequen$) adj3 dental attendance).ti,ab. (5)

112 (clean$ teeth adj2 (irregular$ or regular$ or infrequent$ or frequen$ or never or seldom)).ti,ab. (0)

113 (brush$ teeth adj2 (irregular$ or regular$ or infrequent$ or frequen$ or never or seldom)).ti,ab. (0)

114 ((sweet$ drink$ or fizzy drink$ or sugary snack$ or sweets or confectionery) adj6 (tooth or teeth or dental or oral or caries or decay)).ti,ab. (5)

115 or/103-114 (1226)

116 (letter or editorial or comment reply).dt. (111964)

117 115 not 116 (1170)

118 limit 117 to yr="1990 -Current" (1123)

119 (non-adherence adj2 (patient$ or medication$ or screen$ or treatment or therapy or immunisation or immunization or regimen$ or drug$)).ti,ab. (281)

120 (nonadherence adj2 (patient$ or medication$ or screen$ or treatment or therapy or immunisation or immunization or regimen$ or drug$)).ti,ab. (407)

121 (low adherence adj2 (patient$ or medication$ or screen$ or treatment or therapy or immunisation or immunization or regimen$ or drug$)).ti,ab. (17)

122 (poor adherence adj2 (patient$ or medication$ or screen$ or treatment or therapy or immunisation or immunization or regimen$ or drug$)).ti,ab. (106)

123 ((loss or lack or failure or barrier$ or impediment$ or selective or minimal) adj2 adherence).ti,ab. (378)

124 (non-compliance adj2 (patient$ or medication$ or screen$ or treatment or therapy or immunisation or immunization or regimen$ or drug$)).ti,ab. (165)

125 (noncompliance adj2 (patient$ or medication$ or screen$ or treatment or therapy or immunisation or immunization or regimen$ or drug$)).ti,ab. (500)

126 (low compliance adj2 (patient$ or medication$ or screen$ or treatment or therapy or immunisation or immunization or regimen$ or drug$)).ti,ab. (11)

127 (poor compliance adj2 (patient$ or medication$ or screen$ or treatment or therapy or immunisation or immunization or regimen$ or drug$)).ti,ab. (58)

128 ((loss or lack or failure or barrier$ or impediment$ or selective or minimal) adj2 compliance).ti,ab. (196)

129 treatment refusal/ (584)

130 screening/ (5614)

131 129 and 130 (2)

132 (non-attend$ adj3 screen$).ti,ab. (7)

133 (nonattend$ adj3 screen$).ti,ab. (4)

134 (non-attend$ adj3 appoint$).ti,ab. (27)

135 (nonattend$ adj3 appoint$).ti,ab. (12)

136 (non-attend$ adj3 (check-up$ or checkup$)).ti,ab. (0)

137 (nonattend$ adj3 (check-up$ or checkup$)).ti,ab. (0)

138 (non-attend$ adj3 (mammograph$ or smear test$ or PAP test$ or breast exam$ or CBE)).ti,ab. (2)

139 (nonattend$ adj3 (mammograph$ or smear test$ or PAP test$ or breast exam$ or CBE)).ti,ab. (1)

140 119 or 120 or 121 or 122 or 123 or 124 or 125 or 126 or 127 or 128 or 131 or 132 or 133 or 134 or 135 or 136 or 137 or 138 or 139 (2094)

141 (letter or editorial or comment reply).dt. (111964)

142 140 not 141 (2005)

143 limit 142 to yr="1990 -Current" (1946)

144 safety belts/ (436)

145 (seatbelt$ or seat belt$ or safety belt$).ti,ab. (775)

146 seat restraint$.ti,ab. (3)

147 passenger$ restraint$.ti,ab. (14)

148 driver$ restraint$.ti,ab. (3)

149 ((unbelted or unrestrained) adj2 (driver$ or passenger$)).ti,ab. (24)

150 144 or 145 or 146 or 147 or 148 or 149 (840)

151 safety devices/ or protective devices/ (502)

152 (cycle helmet$ or bike helmet$ or bicycle helmet$).ti,ab. (116)

153 151 or 152 (544)

154 fire prevention/ (88)

155 (smoke adj (alarm$ or sensor$)).ti,ab. (37)

156 (fire adj (alarm$ or sensor$)).ti,ab. (23)

157 154 or 155 or 156 (136)

158 driving under the influence/ (1499)

159 (drink$ adj2 (drive$ or driving)).ti,ab. (961)

160 alcohol impaired driv$.ti,ab. (137)

161 158 or 159 or 160 (1910)

162 150 or 153 or 157 or 161 (3255)

163 (editorial or letter or comment reply).dt. (111964)

164 162 not 163 (3164)

165 limit 164 to yr="1990 -Current" (2853)

166 gambling/ (1943)

167 (gambling or gambler or gamblers).ti,ab. (5292)

168 166 or 167 (5658)

169 (comment reply or editorial or letter).dt. (111964)

170 168 not 169 (5406)

171 limit 170 to yr="1990 -Current" (5175)

172 (17 and 33) or (17 and 41) or (17 and 53) or (17 and 77) or (17 and 94) or (17 and 102) or (17 and 118) or (17 and 143) or (17 and 165) or (17 and 171) (6933)

173 (33 and 17) or (33 and 41) or (33 and 53) or (33 and 77) or (33 and 94) or (33 and 102) or (33 and 118) or (33 and 143) or (33 and 165) or (33 and 171) (13373)

174 (41 and 17) or (41 and 33) or (41 and 53) or (41 and 77) or (41 and 94) or (41 and 102) or (41 and 118) or (41 and 143) or (41 and 165) or (41 and 171) (13773)

175 (53 and 17) or (53 and 33) or (53 and 41) or (53 and 77) or (53 and 94) or (53 and 102) or (53 and 118) or (53 and 143) or (53 and 165) or (53 and 171) (47329)

176 (77 and 17) or (77 and 33) or (77 and 41) or (77 and 53) or (77 and 94) or (77 and 102) or (77 and 118) or (77 and 143) or (77 and 165) or (77 and 171) (3571)

177 (94 and 17) or (94 and 33) or (94 and 41) or (94 and 53) or (94 and 77) or (94 and 102) or (94 and 118) or (94 and 143) or (94 and 165) or (94 and 171) (47282)

178 (102 and 17) or (102 and 33) or (102 and 41) or (102 and 53) or (102 and 77) or (102 and 94) or (102 and 118) or (102 and 143) or (102 and 165) or (102 and 171) (79)

179 (118 and 17) or (118 and 33) or (118 and 41) or (118 and 53) or (118 and 77) or (118 and 94) or (118 and 102) or (118 and 143) or (118 and 165) or (118 and 171) (245)

180 (143 and 17) or (143 and 33) or (143 and 41) or (143 and 53) or (143 and 77) or (143 and 94) or (143 and 102) or (143 and 118) or (143 and 165) or (143 and 171) (486)

181 (165 and 17) or (165 and 33) or (165 and 41) or (165 and 53) or (165 and 77) or (165 and 94) or (165 and 102) or (165 and 118) or (165 and 143) or (165 and 171) (1759)

182 (171 and 17) or (171 and 33) or (171 and 41) or (171 and 53) or (171 and 77) or (171 and 94) or (171 and 102) or (171 and 118) or (171 and 143) or (171 and 165) (4271)

183 172 or 173 or 174 or 175 or 176 or 177 or 178 or 179 or 180 or 181 or 182 (63941)

184 double-blind.tw. (13750)

185 random$ assigned.tw. (18337)

186 control.tw. (222575)

187 184 or 185 or 186 (244674)

188 183 and 187 (9254)

189 trial.ti. (13244)

190 (evaluate$ or evaluation or evaluating).ti. (36745)

191 Intervention/ (27354)

192 evaluation study.ab. (613)

193 (intervention or program or programme).ti. (49212)

194 interrupted time series.ti,ab. (335)

195 (before-after adj2 study).ti,ab. (60)

196 (before adj3 study).ti,ab. (1163)

197 experimental study.ti,ab. (4247)

198 quasi-experimental study.ti,ab. (919)

199 quasi experimental study.ti,ab. (919)

200 (pre post or pre-post).ti,ab. (2762)

201 189 or 190 or 191 or 192 or 193 or 194 or 195 or 196 or 197 or 198 or 199 or 200 (116491)

202 183 and 201 (5289)

203 188 or 202 (13013)

204 Communities/ (16750)

205 (community or community-based).ti,ab. (128499)

206 Neighborhoods/ (3829)

207 (neighbourhood$ or neighborhood$).ti,ab. (10622)

208 Urban Environments/ (13493)

209 urban communit$.ti,ab. (1366)

210 Rural Environments/ (9373)

211 rural communit$.ti,ab. (2355)

212 ((disadvantaged or poor or deprived) adj (communit$ or area$)).ti,ab. (984)

213 (work or worksite or workplace).ti,ab. (251850)

214 Working Conditions/ (13618)

215 (web-based or web or website or online or internet or computer or computer-tailored or computer-based or online or email or telephone).ti,ab. (98209)

216 Internet/ or Telephone Systems/ or Computer Mediated Communications/ (20445)

217 ((parent$ or family or women$ or woman$ or sure start) adj2 (centre$ or center$ or co-op or cooperative or clinic$)).ti,ab. (5994)

218 (GP practice$ or general practice or family practice or primary care).ti,ab. (19777)

219 General Practitioners/ or Family Physicians/ (4816)

220 ((emergency or outpatient) adj (department$ or clinic$ or ward$)).ti,ab. (7328)

221 (accident adj emergency).ti,ab. (20)

222 (campus$ or college$ or classroom$).ti,ab. (108780)

223 (church$ or home$ or home-based or pharmacy or pharmacies or night club$ or beer hall$).ti,ab. (90039)

224 204 or 205 or 206 or 207 or 208 or 209 or 210 or 211 or 212 or 213 or 214 or 215 or 216 or 217 or 218 or 219 or 220 or 221 or 222 or 223 (627627)

225 203 and 224 (4301)

226 (lifestyle adj2 (intervention$ or program$)).ti,ab. (757)

227 (life style adj2 (intervention$ or program$)).ti,ab. (27)

228 (behavior$ change adj (intervention$ or program$)).ti,ab. (337)

229 (behaviour$ change adj (intervention$ or program$)).ti,ab. (84)

230 (multiple risk factor adj2 (program$ or intervention$)).ti,ab. (26)

231 (multifactorial lifestyle adj (intervention$ or program$)).ti,ab. (2)

232 (health behavior$ adj (program$ or intervention$)).ti,ab. (89)

233 (health behaviour$ adj (program$ or intervention$)).ti,ab. (11)

234 multiple health behavior$ change intervention$.ti,ab. (2)

235 multiple health behaviour$ change intervention$.ti,ab. (1)

236 multiple behavior$ risk factor$ intervention$.ti,ab. (1)

237 multiple behaviour$ risk factor$ intervention$.ti,ab. (0)

238 multiple behavior$ risk factor$ program$.ti,ab. (0)

239 multiple behaviour$ risk factor$ program$.ti,ab. (0)

240 multiple risk behaviour$ intervention$.ti,ab. (0)

241 multiple risk behavior$ intervention$.ti,ab. (1)

242 multiple risk behaviour$ program$.ti,ab. (0)

243 multiple risk behavior$ program$.ti,ab. (0)

244 226 or 227 or 228 or 229 or 230 or 231 or 232 or 233 or 234 or 235 or 236 or 237 or 238 or 239 or 240 or 241 or 242 or 243 (1303)

245 225 or 244 (5502)

246 limit 245 to yr="1990 -Current" (5475)

**Science Citation Index**

**Via Web of Science**

**Search date 17^th^ January 2013**

**7048 records identified**

| # 4 | [**7,048**](http://apps.webofknowledge.com/summary.do?product=WOS&doc=1&qid=16&SID=Q25mahb9C97bE91NbDn&search_mode=CombineSearches) | #3 OR #2 OR #1  *Databases=SCI-EXPANDED Timespan=1990-02-01 - 2013-01-17*  *Lemmatization=Off* |
| --- | --- | --- |
| # 3 | [**5**](http://apps.webofknowledge.com/summary.do?product=WOS&doc=1&qid=11&SID=Q25mahb9C97bE91NbDn&search_mode=AdvancedSearch) | TS=("multiple health behavior* change intervention*") OR TS=("multiple health behaviour* change intervention*") OR TS=("multiple behavior* risk factor* intervention*") OR TS=("multiple behaviour* risk factor* intervention*") OR TS=("multiple behavior* risk factor* program*") OR TS=("multiple behaviour* risk factor* program*") OR TS=("multiple risk behaviour* intervention*") OR TS=("multiple risk behavior* intervention*") OR TS=("multiple risk behaviour* program*") OR TS=("multiple risk behavior* program*")  *Databases=SCI-EXPANDED Timespan=1990-02-01 - 2013-01-17*  *Lemmatization=Off* |
| # 2 | [**1,289**](http://apps.webofknowledge.com/summary.do?product=WOS&doc=1&qid=7&SID=Q25mahb9C97bE91NbDn&search_mode=GeneralSearch) | Topic=("multiple risk factor" NEAR/2 program*) OR Topic=("multiple risk factor" NEAR/2 intervention*) OR Topic=("multifactorial lifestyle" NEAR intervention*) OR Topic=("multifactorial lifestyle" NEAR program*) OR Topic=("health behavior*" NEAR program*) OR Topic=("(health behavior*" NEAR intervention*) OR Topic=("health behaviour*" NEAR program*) OR Topic=("health behaviour*" NEAR intervention*)  *Databases=SCI-EXPANDED Timespan=1990-02-01 - 2013-01-17*  *Lemmatization=Off* |
| # 1 | [**6,031**](http://apps.webofknowledge.com/summary.do?product=WOS&doc=1&qid=15&SID=Q25mahb9C97bE91NbDn&search_mode=AdvancedSearch) | TS=(lifestyle NEAR/2 intervention*) OR TS=(lifestyle NEAR/2 program*) OR TS=("life style" NEAR/2 intervention*) OR TS=("life style" NEAR/2 program*) OR TS=("behavior* change" NEAR intervention*) OR TS=("behavior* change" NEAR program*) OR TS=("behaviour* change" NEAR intervention*) OR TS=("behaviour* change" NEAR program*)  *Databases=SCI-EXPANDED Timespan=1990-02-01 - 2013-01-17*  *Lemmatization=Off* |
